# Acute Effects of Nutritive and Non-Nutritive Sweeteners on Postprandial Blood Pressure

**DOI:** 10.3390/nu11081717

**Published:** 2019-07-25

**Authors:** Hung Pham, Liza K. Phillips, Karen L. Jones

**Affiliations:** 1 NHMRC Centre of Research Excellence in Translating Nutritional Science to Good Health, Adelaide Medical School, The University of Adelaide, Adelaide, SA 5000, Australia; 2 Endocrine and Metabolic Unit, Royal Adelaide Hospital, Adelaide, SA 5000, Australia

**Keywords:** postprandial hypotension, nutritive sweeteners, non-nutritive sweeteners, glucose, fructose, sucrose, d-xylose, xylitol, erythritol, maltose, maltodextrin, tagatose

## Abstract

Postprandial hypotension (PPH) is under-recognised, but common, particularly in the elderly, and is of clear clinical importance due to both the independent association between PPH and an increase in mortality and lack of effective management for this condition. Following health concerns surrounding excessive consumption of sugar, there has been a trend in the use of low- or non-nutritive sweeteners as an alternative. Due to the lack of literature in this area, we conducted a systematic search to identify studies relevant to the effects of different types of sweeteners on postprandial blood pressure (BP). The BP response to ingestion of sweeteners is generally unaffected in healthy young subjects, however in elderly subjects, glucose induces the greatest decrease in postprandial BP, while the response to sucrose is less pronounced. The limited studies investigating other nutritive and non-nutritive sweeteners have demonstrated minimal or no effect on postprandial BP. Dietary modification by replacing high nutritive sweeteners (glucose, fructose, and sucrose) with low nutritive (d-xylose, xylitol, erythritol, maltose, maltodextrin, and tagatose) and non-nutritive sweeteners may be a simple and effective management strategy for PPH.

## 1. Introduction

The consumption of nutritive sweeteners has dramatically increased worldwide due to increasing urbanisation and beverage promotion [1]. Nutritive sweeteners commonly found in foods and beverages include glucose, fructose and sucrose [2,3]. More recently, some other sweeteners like d-xylose, xylitol, erythritol, maltose, maltodextrin, stevia and tagatose with lower energy have been used [2,3]. There has been a long history of debate as to the detrimental health effects of sucrose, glucose, and more recently, fructose, particularly when consumed in excess. The effects of both nutritive and non-nutritive sweeteners on cardiovascular risk factors, including blood pressure (BP), have been extensively investigated, however, less is known about their specific effects in the postprandial state. Postprandial hypotension (PPH), a fall in systolic BP (SBP) ≥20 mmHg after a meal, is now recognized as a frequent and important clinical problem, with a reported prevalence of 24–48% in the healthy elderly [4]. PPH is particularly common in conditions associated with autonomic dysfunction e.g., in type 2 diabetes mellitus (T2DM) (~40%) [4,5] and Parkinson’s disease (PD) (40–100%) [4,5]. Patients with PPH are at increased risk of syncope and falls [6], and importantly the presence of PPH has been established as an independent predictor of increased mortality [7]. In a cohort study of 401 elderly ambulatory hypertensive subjects, 292 (72.8%) were found to have PPH, and over a 4 year period, 34 died from cardiovascular disease; the post-breakfast BP fall was the strongest predictor of mortality in this cohort [8]. In a 29-month prospective study of 499 nursing home residents, the maximum fall in postprandial BP was shown to be an independent predictor of subsequent falls, syncope, new cardiovascular events (myocardial infarction and stroke) and total mortality [9].

**Figure 1:**
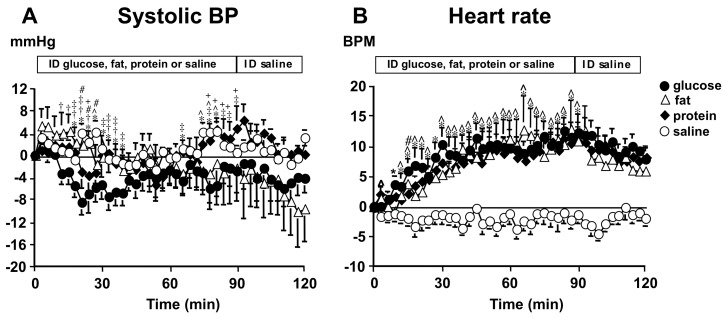
Changes from baseline in (**A**) Systolic blood pressure (BP) and (**B**) Heart rate in eight healthy older participants in response to intraduodenal (ID) infusion of glucose, fat, protein, and saline. Values are means ± SEM. * *p* < 0.05 for glucose compared with saline; † *p* < 0.05 for glucose compared with fat; ‡ *p* < 0.0001 for glucose compared with protein; ^ *p* < 0.05 for fat compared with saline; # *p* < 0.05 for protein compared with saline; + *p* < 0.05 for fat compared with protein [14]. BPM = beats per minute.

The pathophysiology of PPH is multifactorial and incompletely understood, however, it involves the interplay between autonomic and neural mechanisms, including the release of gut hormones, which are influenced by meal composition, gastric distension, and small intestinal nutrient delivery [4]. After a meal, there is a doubling of the superior mesenteric artery (SMA) blood flow [5] and in healthy young individuals with intact baroreflex function, the increase in splanchnic blood flow is accompanied by concomitant increases in heart rate (HR), peripheral vascular resistance, stroke volume and cardiac output [10]. In patients with PPH, these compensatory responses are inadequate [5]. The postprandial fall in BP is greater when gastric emptying is more rapid [11,12,13], whereas gastric distension attenuates the fall in BP in both healthy young and older participants [11,12,13]. The macronutrient composition of the meal is also an important determinant of postprandial BP, with carbohydrate inducing an earlier fall in BP compared with protein and fat [14] (Figure 1). The interaction between nutrients and the small intestine stimulates the secretion of the incretin hormones, glucagon-like peptide-1 (GLP-1) from L-cells and glucose-dependent insulinotropic polypeptide (GIP) from K-cells [15]. GLP-1 stimulates insulin secretion, inhibits glucagon secretion and slows gastric emptying [16,17]. Acute infusion of GLP-1 has been shown to attenuate the fall in BP and increases SMA blood flow following oral [16] or intraduodenal (ID) glucose [16,18]. In contrast, exogenous GIP has been shown to increase the rate of gastric emptying [19], although results have been conflicting [20] and there have been no studies directly investigating the effect of GIP on postprandial BP. A reduction in BP is not observed following intravenous administration of glucose [21], which is a substantial stimulus to insulin secretion, suggesting that insulin by itself if not an important mediator of PPH. Furthermore, PPH is seen in the absence of insulin secretion i.e., in those with type 1 diabetes [22].

In recent years, due to several negative health effects from the excessive consumption of added sugar, a variety of non-nutritive artificial sweeteners have increasingly been used in the food industry as an alternative to sugar among people of all ages, particularly directed to the obese and those with diabetes, with the aim of reducing energy intake and minimising the risk of obesity, diabetes and cardiovascular disease [3,23]. Beverages account for the highest percentage of non-nutritive sweetener utilisation in the world [24,25]. In the United States, non-nutritive sweeteners were used in up to 32% and 19% of beverages consumed by adults and children respectively from 2007 to 2010 [26]. In 2018, the American Heart Association suggested that short-term use of “non-nutritive” sweeteners instead of “nutritive sweeteners” can reduce caloric intake in the management of excess weight and obesity [27].

There are some differences between regulatory bodies regarding approval of nutritive and non-nutritive sweeteners. The six non-nutritive sweeteners approved by Food and Drug Administration (FDA) as food additives in the United States include sucralose, Acesulfame-K, aspartame, saccharin, neotame and advantame [2,28,29]. Stevia [29,30] has not received official FDA approval but has been rated as generally recognized as safe (GRAS) and is being increasingly used as a non-nutritive sweetener [31]. Maltitol similarly has not received official FDA approval, but may be used in food manufacture [31]. The nutritive sweeteners currently approved by the FDA include mannitol, xylitol, sorbitol and erythritol [32]. The European Food Safety Authority (EFSA) has approved the non-nutritive sweeteners Acesulfame-K, aspartame, cyclamate, neohesperidin dihydrochalcone, saccharin, sucralose, neotame, thaumatin and stevioside, while approved nutritive sweeteners include erythritol, isomalt, lactitol, maltitol, mannitol, sorbitol and xylitol [31].

There have been a number of short-term studies evaluating the physiological responses to nutritive and non-nutritive sweeteners that have included assessment of BP. With the increasing widespread use of non-nutritive sweeteners and the documented severe clinical sequelae of PPH, it is clearly important to ascertain the extent to which these agents decrease postprandial BP. To date, a published systematic summary of the impact of nutritive and non-nutritive sweeteners on postprandial BP has not been performed. For this systematic review, we aim to establish current scientific evidence of the effects of some of the most commonly used FDA-regulated nutritive and non-nutritive sweeteners on postprandial BP, as well as their potential applications in the management of PPH. 

## 2. Approach

Based on the pathophysiology of PPH, a systematic review of the PubMed database was conducted up to March 2019 to identify articles related to the effects of different types of sweeteners on postprandial BP. The following keywords were used: “sweeteners” or “nutritive sweeteners” or “caloric sweeteners” or “sucrose” or “fructose” or “glucose” or “xylose” or “xylitol” or “erythritol” or “maltose” or “maltodextrin” or “tagatose” or “non-nutritive sweeteners” or “non-caloric sweeteners” or “sucralose” or “Acesulfame-K” or “aspartame” or “saccharin” or “stevia” or “steviol glycoside” or “neotame” or “advantame”) and (“postprandial blood pressure” or “postprandial hypotension” or “hypotensive response”).

Screening of studies was performed initially by assessment of the relevance of the abstract by two independent reviewers. The reference lists of relevant articles were also reviewed. Study inclusion was based on the guidelines for preferred reporting items for systematic reviews and meta-analyses (PRISMA) [33].

## 3. Results

**Figure 2:**
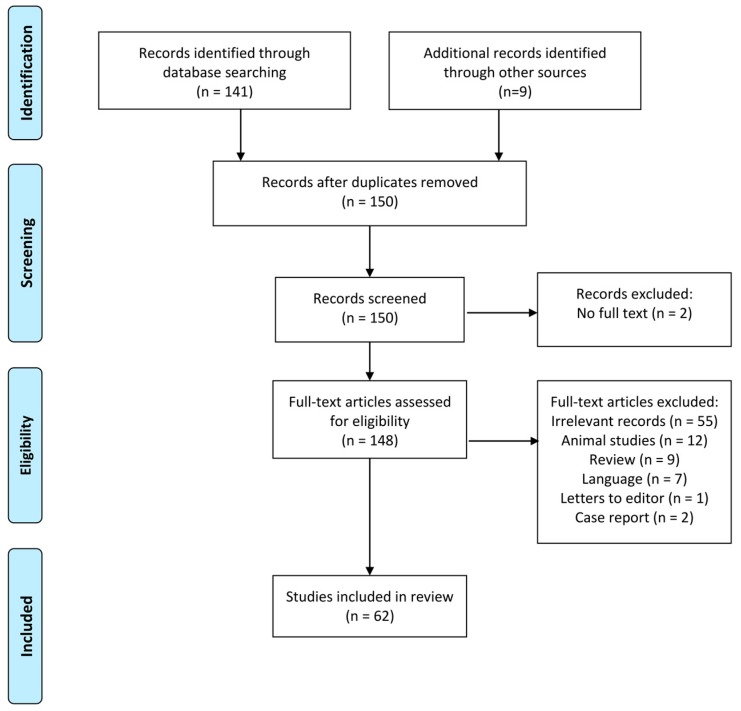
Flow diagram for the selection of studies for review based on the preferred reporting items for systematic reviews and meta-analyses (PRISMA) 2009 statement.

A total of 150 papers were identified from the database search. There were no duplicate papers. Of the 150 papers, papers without full text (*n* = 2), irrelevant papers (*n* = 55), animal studies (*n* = 12), reviews (*n* = 9), papers that were unavailable in the English language (*n* = 7), letters to the editor or comments (*n* = 1), and case reports (*n* = 2) were removed, leaving a total of 62 papers which were reviewed. Figure 2 summarizes the selection process.

## 4. Nutritive Sweeteners

### 4.1. Glucose 

Glucose is the most abundant monosaccharide and is synthesised from water and carbon dioxide through photosynthesis and concentrated to create starch [34]. Foods containing relatively higher proportions of naturally occurring glucose include some fruits, such as grapes and bananas (~6–8%), while the glucose content of honey may approach ~38% [35,36]. It is an essential energy source for the red blood cells and brain, although the latter can also utilise ketone bodies [2,37]. Ingested complex carbohydrates are hydrolysed to monosaccharides before being absorbed [37]. The majority of studies investigating PPH have utilised an oral [11,12,13,16,38,39,40,41,42,43,44,45,46,47,48,49,50,51,52,53,54,55,56,57,58,59,60,61,62,63,64,65,66,67,68,69,70,71,72,73], and/or ID glucose [14,18,74,75,76,77,78,79,80,81,82,83,84,85,86,87,88] load that induces a substantial fall in BP. There have been a total of 58 studies related to effects of glucose on postprandial BP in a range of cohorts including health [11,12,14,16,18,39,40,41,42,43,46,48,49,50,51,52,54,55,56,58,60,62,63,64,65,66,68,69,70,71,74,75,77,78,79,80,81,82,83,84,86,87,89], PPH [48,81], diabetes [11,13,16,38,57,64,67,68,69,76,85,88], autonomic failure [43,45,56,57,58], PD [47,57,71,72], hypertension [53,59,60,61,62,66], multiple system atrophy (MSA) [56,57,58,70] and other conditions [41,44,45,53,65,80] (Table 1). Intensive care unit (ICU) survivors are more predisposed to PPH; a marked and prolonged decrease in SBP and diastolic BP (DBP) was observed in 35 older patients discharged from the ICU [44], 10 of whom experienced PPH [44]. In healthy older participants [11,12,46] and patients with T2DM [11,64], the fall in BP following glucose ingestion has been shown to be related to the rate of gastric emptying of glucose, i.e., faster gastric emptying is associated with a greater fall in BP. In contrast, in healthy young participants, there is little fall, and in some cases an increase in, SBP following oral glucose [53,89]. The fall in postprandial BP appears to be on a continuum so that with increasing age, the fall in BP is greater. In a study by our group, following a 75 g glucose drink in 300 mL water, there was a fall in SBP and a rise in HR in both healthy older participants and people with PPH compared with iso-volaemic water [48], however, the maximum postprandial fall in SBP was greater in the participants with PPH [48].

#### 4.1.1. Intraduodenal Glucose Infusion

When the protective mechanism of gastric distension [86,87] is bypassed by infusing glucose directly into the duodenum, the fall in BP is greater compared to oral glucose in healthy older participants [54]. Vanis et al. showed that gastric distension, by as little as 100 mL water, could mitigate the fall in BP induced by ID glucose in healthy older participants, supporting the potential use of non-nutritive gastric distension in the management of PPH [87]. ID glucose-induced reductions in BP are observed in both older people [54,75,81] and patients with T2DM [76,85,88]. The hypotensive response to ID glucose depends primarily on the small intestinal glucose load, but solution concentration does not appear to be important [74]. Within the normal physiological range of gastric emptying (1–4 kcal/min) [90], the rate of small intestinal delivery correlates with the magnitude of the fall in BP [76,77,79]. Trahair et al. reported that SBP decreased significantly during 2 kcal/min and 3 kcal/min glucose infusions, but not during 1 kcal/min glucose infusion; indicating that a threshold for the fall occurs between 1–2 kcal/min [91]. On the contrary, young healthy participants with sufficient cardiovascular responses remain normotensive [79] or become only slightly hypotensive [81] after ID glucose infusion. There is no difference in postprandial BP following ID glucose infusion between young healthy and obese groups [80]. 

#### 4.1.2. Management of PPH

Due to its ability to induce a prominent hypotensive response, several studies investigating non-pharmacological [63,67,68,84,86,87] and pharmacological [18,55,57,58,59,60,61,64,65,83,85,88] approaches to the management of PPH have used glucose as test meals. Interventions based on slowing gastric emptying [63,84] and small intestinal absorption of nutrients [82] attenuate the postprandial fall in BP. Guar gum, a vegetable-based, gel-forming non-absorbable carbohydrate, commonly used as a bulking agent, delays gastric emptying, slows the intestinal absorption of glucose and, accordingly, attenuates the magnitude of the fall in BP after oral and ID glucose [13,63,84]. Intermittent walking may also be effective in preventing PPH induced by glucose ingestion [67,68]. In terms of the pharmacologic management of PPH, numerous medications have been tested. One study has shown that maximal postprandial fall in sitting SBP after a standardized 400 kcal glucose drink is attenuated by caffeine [55], although the benefits of caffeine as a treatment for PPH are inconsistent and largely empirical. The effects of the somatostatin analogue, octreotide, in attenuating the fall in BP after oral glucose has been demonstrated in three studies [58,60,61]. These effects are not mediated via the inhibition of insulin secretion [61]. Voglibose, an alpha-glucosidase inhibitor, is effective in attenuating PPH in neurologic patients [65]. More recently, GLP-1 based therapy has drawn more attention. In a placebo-controlled study, intravenous GLP-1 infusion (0.9 pmol kg^−1^.min^−1^) in 14 healthy older individuals and 10 patients with T2DM, resulted in the slowing of gastric emptying and attenuation in the fall in BP following ingestion of a radiolabelled 75 g glucose drink [16]. GLP-1 also reduces the maximum fall in SBP in response to ID glucose [18]. Both short-acting GLP-1 receptor agonists, exenatide [85] and lixisenatide [64], known to slow gastric emptying, markedly attenuate the decrease in BP compared to placebo. In contrast, the dipeptidyl peptidase-4 inhibitor (DPP-4), vildagliptin, known to block the degradation of the incretin hormones GLP-1 and GIP, did not attenuate the fall in SBP and DBP during ID glucose administration compared with placebo in T2DM [88]. Recent evidence suggests that GIP may have detrimental effects to lower postprandial BP and it is possible that the beneficial effects of GLP-1 by DPP-4 inhibition were nullified by GIP. The effect of the anti-diabetic medication, metformin, has also been evaluated in two studies [38,88], but the outcomes are inconsistent and more studies are warranted. Other investigators have reported that the antihypertensive, nitrendipine [59], or a combination of selective α1 and β1-adrenergic agonists [57], might attenuate the postprandial fall in BP after glucose ingestion, while the antiemetic drug, granisetron, a 5-HT3 antagonist, [83] has no benefit in the management of PPH.

### 4.2. Fructose

Fructose, a monosaccharide found in fruit, honey, and some vegetables [2], may be preferred over glucose by consumers and cooks due to its intrinsically greater sweetness and ability to improve the appearance and texture of baked goods [92]. Fructose has been used increasingly as an alternative to glucose or sucrose [93,94,95] in several processed foods and beverages, especially in diets targeting patients with T2DM, as it provides fewer calories for the same level of sweetness as glucose [96,97]. In contrast to glucose, the metabolism of fructose primarily occurs in the liver, entering cells in an insulin-independent fashion [98]. Fructose is not an insulin secretagogue [98], inducing a substantially lower glycaemic response compared with glucose [95]. However, although there is no clear consensus in the literature, the potential for increased de novo lipogenesis from excessive fructose intake, particularly in those with insulin resistance, has raised the concern regarding the widespread use of this monosaccharide [99]. Compared with glucose, fructose is absorbed more slowly from the intestine [100,101]. There has been a total of five studies relating to the effects of oral fructose on postprandial BP in a range of cohorts including in healthy participants [39,40,41,50,89] and hypertensive patients [41] (Table 2).

**Figure 3:**
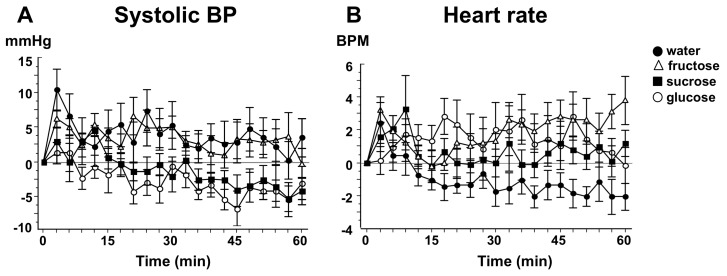
Change from baseline in (**A**) Systolic blood pressure (BP) and (**B**) Heart rate in 10 healthy older people following ingestion of four different study drinks (○, glucose; ■, sucrose; ∆, fructose; ●, water). Values are means ± SEM [50].

Some studies have demonstrated no change in BP in healthy young [41] and older participants [50] as well as those with hypertension [41] following ingestion of fructose. In healthy older people, SBP decreased significantly from baseline following glucose: −3.96 ± 1.38 mmHg but not after the fructose drink: 2.59 ± 1.62 mmHg [41] (Figure 3). Two other studies reported an increase in BP in healthy young participants following ingestion of fructose [39,40]. This may be due to the slower intestinal absorption rate of fructose and lower glycaemic response compared with glucose [102,103], which may also result in a limited increase in SMA blood flow. However, there have been no studies comparing the difference in postprandial intestinal vascular perfusion following oral glucose and fructose. In addition, due to the lower insulinotropic effects of fructose compared with glucose and sucrose [104], effects on total vascular peripheral resistance may be mitigated [39,89].

### 4.3. Sucrose 

Sucrose is a common table sugar produced naturally in fruit and vegetables and often added to many processed foods [108]. Sucrose tastes sweeter than glucose, but not as sweet as fructose. The digestion of sucrose is initiated in the mouth and stomach, but the majority of this disaccharide is hydrolysed and digested in the small intestine by the α-glucosidase sucrase to release an equimolar mixture of glucose and fructose [109]. There have been five studies relating to effects of sucrose on postprandial BP in healthy participants [40,50,105,106,107] (Table 2): four used an oral load [40,50,105,107] while one study used an ID infusion [106].

The cardiovascular effects of sucrose are more similar to those observed following glucose than fructose, despite its equimolar constitution [110]. Visvanathan et al. reported that ingestion of 50 g sucrose or 50 g glucose induced a comparable fall in SBP in healthy elderly individuals, but the decrease in SBP occurred earlier after glucose, than sucrose, ingestion [50] (Figure 3). This may be due to the fact that sucrose is a disaccharide, requiring hydrolysis prior to mediating cardiovascular effects [50]. In comparison, a 60 g sucrose drink had no substantial impact on BP in healthy young participants [40], which may be due to the more effective postprandial haemodynamic responses compared with an older population. The α-glucosidase inhibitor, acarbose, is approved for the treatment of T2DM and is known to reduce postprandial glycaemia [111] through stimulation of GLP-1 [112], and slowing of gastric emptying [105,113]. In healthy older participants, acarbose substantially decreases falls in SBP and DBP induced by not both oral [105], and ID [106] sucrose, loads. 

### 4.4. d-Xylose and Xylitol 

Xylose, a monosaccharide of the aldopentose type consists of five carbon atoms and is derived from wood. The free aldehyde group reduces its sweetness and makes it a widely used alternative for glucose in the diabetic diet. Xylose is primarily absorbed by passive penetration through the wall of human duodenum and jejunum [114], with the remainder delivered to the ileum and the colon. Xylose is emptied from the stomach at a similar rate to glucose [49]. There have been three studies related to effects of oral xylose on postprandial BP in healthy older participants [43,45,49], orthostatic hypotension (OH) [45] and autonomic failure [43,45] patients (Table 2). While Mathias et al. [43] reported xylose induced a milder and more transient fall in postprandial BP compared with glucose in those with chronic autonomic failure, Robinson et al. reported that glucose and xylose ingestion induced a comparable fall in SBP at 60–90 min in supine patients diagnosed with OH and autonomic failure [45]. In a previous study by our group, designed to compare effects of glucose and xylose on BP, gastric emptying and incretin hormones, xylose emptied from the stomach at a comparable rate to glucose, with a greater and more prolonged effect on GLP-1 secretion in healthy older participants [49]. However, there was no change in postprandial BP observed, potentially due to the effects of GLP-1 in delaying the nutrient absorption in the distal small intestine. These findings support the replacement of glucose by xylose as a simple management to decrease the postprandial fall in BP [49].

Reduction of xylose by catalytic hydrogenation produces xylitol [115]. Xylitol has a comparable sweetness to sucrose and contains less than one-third of calories in conventional sugars. As xylitol is metabolized in an insulin-independent manner, its use has been widely advocated in the prevention and control of hyperglycaemia, obesity, and related metabolic disorders [116,117]. However, xylitol is often associated with diarrhoea due to its stimulatory effect on the intestinal transit and the release of plasma motilin [118]. Salminen et al. [118] reported that 30 g xylitol solution was emptied from the stomach at a significantly slower rate than a 30 g of glucose, suggesting that xylitol might protect against PPH, although further studies are required.

### 4.5. Erythritol

Erythritol, a four-carbon pentose, is made through food fermentation but may occur naturally in a wide variety of fruits and mushrooms. Erythritol has a sweetness of ~70% compared with sucrose and has minimal insulinotropic effects [119,120]. Nevertheless, the effect erythritol on BP has not been studied. In patients with T2DM, acute consumption of erythritol has vasoprotective effects by decreasing oxidative stress and endothelial dysfunction [121].

### 4.6. Maltose and Maltodextrin 

Maltose, or malt sugar, a disaccharide composed of two glucose units, is an intermediate in the intestinal breakdown of glycogen and starch and is found in germinating grains [122]. Maltodextrin is a nutritive saccharide polymer consisting of D-glucose units with a low sweetness (dextrose equivalency of less than 20). It is often used in the production of soft drinks, candy, and processed food [123]. Both maltose [124] and maltodextrin [125] are emptied more slowly from the stomach compared with sucrose, although there is no significant difference in gastric emptying between maltodextrin and glucose [125]. To our knowledge, there have been no studies investigating the effects of maltose on postprandial BP. There has been only one study related to effects of oral maltodextrin on postprandial BP in healthy older participants [107] (Table 2) in which postprandial MAP and DBP were shown to be greater after sucrose compared to after maltodextrin [107]. The underlying mechanisms remain unclear [107]. 

### 4.7. Tagatose 

Tagatose has a very similar structure to sucrose, is commonly found in dairy products [126] and is low calorie; only 20 to 25% of ingested D-tagatose is absorbed through the small intestine to provide 1.5 kcal/g [127]. The remaining ingested tagatose is fermented into short-chain fatty acids by colonic bacteria [2,128]. Tagatose has a substantial hypoglycaemic effect without major adverse effects, contributing to its potential therapeutic benefits in obese people and patients with T2DM [129,130,131,132]. While tagatose has been shown to slow gastric emptying [133,134], to our knowledge there is no information regarding the effect of tagatose on BP. 

## 5. Non-Nutritive Sweeteners

### 5.1. Sucralose

Sucralose, a noncaloric artificial sweetener with a sweetness approximately 600 times higher than that of sucrose [135], is increasingly being used as a sugar substitute to reduce calorie intake, especially in obese people and those with diabetes [3]. It is almost unabsorbed in the small intestine and excreted in the faeces in both animal [136,137] and human [138] experiments. In healthy participants, sucralose empties at a similar rate to saline [139].

**Figure 4:**
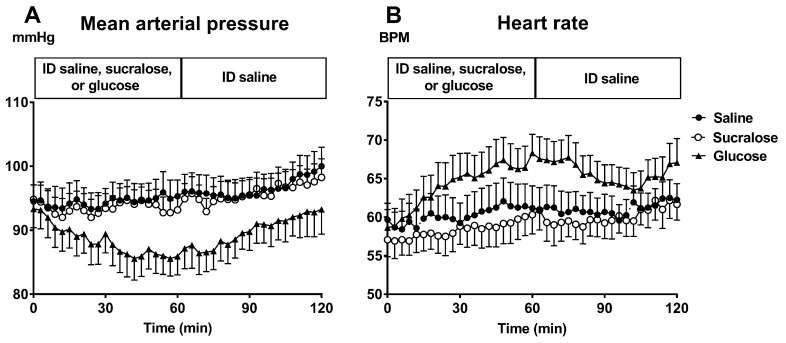
(**A**) Mean arterial pressure (MAP) and (**B**) Heart Rate in 12 healthy older subjects after intraduodenal (ID) infusion of glucose (▲), sucralose (ο), and saline (•). Values are means ± SEM. MAP: *p* < 0.05 for glucose compared with sucralose or saline, and *p* = 1.0 for sucralose compared with saline. Heart rate: *p* < 0.05 for glucose compared with sucralose, *p* = 0.07 for glucose compared with saline, and *p* = 1.0 for sucralose compared with saline [78].

There have been two studies investigating effects of sucralose on postprandial BP [78,140] (Table 3). Kazmi et al. reported that there was no change in BP following oral sucralose in 200 young healthy participants compared to saline. In a recent study by our group, while ID infusion of glucose at a rate of 3 kcal/min induced a substantial fall in postprandial BP and increase in SMA blood flow in 12 healthy older participants, there were no significant effects on BP or splanchnic blood flow following an ID infusion of sucralose [78] (Figure 4). Studies by others have shown that sucralose has no effect on insulin, GLP-1 or GIP secretion or SMA blood flow [139,141], supporting a therapeutic role for sucralose in the dietary management of PPH.

### 5.2. Acesulfame-K

Acesulfame-K, a non-nutritive sweetener, was invented in 1967 by the pharmaceutical company, Hoechst AG. It is estimated to be 200 times sweeter than sucrose and is often mixed with sucralose or aspartame in foods and beverages [28]. In 2009, Brown et al. reported that the GLP-1 release increased by more than 30% after drinking “diet soda”, comprising sucralose, aspartame and Acesulfame-K compared to carbonated water [142]. However, this finding was not replicated in subsequent studies [141,143]. Acesulfame-K alone or combined has no effect on GLP-1 secretion, insulin, or blood glucose. There has been only one study investigating the effect of Acesulfame-K on BP [140] (Table 3). Kazmi et al. reported that SBP was lower at 60 min following the ingestion of 3.24 gm (45 mg/kg) of Acesulfame-K in 50 healthy young participants compared to controls. The design of that study was, however, suboptimal, as SBP and DBP were only measured at 30, 60, 90 and 120 min and the baseline measurement was not taken into account.

### 5.3. Aspartame 

Aspartame, a methyl ester of aspartic acid and phenylalanine dipeptide, was approved as a non-nutritive sweetener in 1996 by the FDA. It is 160 to 220 times sweeter than sucrose and the only non-nutritive sweetener providing energy (4 kcal/g). However, due to the intense sweetness, only a tiny quantity is used in foods and soft drinks and the number of calories is negligible, hence, it is considered ‘non-nutritive’ [2,3]. Little et al. [144] demonstrated that the gastric emptying of aspartame does not differ from that of water. There has been only one study investigating the effect of aspartame on BP [140] (Table 3). In this study, young healthy participants experienced falls in SBP at 60, 90 and 120 min following the ingestion of 10.8 g (150 mg/kg) aspartame compared to 10 g of cellulose (control) (109.82 ± 11.39 vs. 116.92 ± 8.98, 112.54 ± 11.18 vs. 117.84 ± 10.2 and 112.02 ± 10.54 vs. 117.16 ± 8.62 respectively) [140]. Again, the design of that study was, suboptimal, as SBP and DBP were only measured at 30, 60, 90 and 120 min and the baseline measurement was not taken into account.

### 5.4. Saccharin

Saccharin is the oldest non-nutritive sweetener that is currently approved, is not metabolized in humans and is considered non-carcinogenic [2,3]. To date, there are no studies investigating the acute effects of saccharin on BP. 

### 5.5. Steviol Glycoside

Steviol glycoside sweeteners are extracted and purified from the leaves of the Stevia rebaudiana Bertoni plant, which is native to South America. Its sweet potency is reported to be 200 to 400 times sweeter than table sugar [145,146]. Steviol glycoside has been categorized as a non-caloric sweetener and widely used in the food and soft drinks consumed by millions of people and is well tolerated [126]. In one study, postprandial blood glucose and insulin levels were significantly lower when healthy lean and obese participants consumed a steviol glycoside preload before meals compared with an isocaloric sucrose preload [147]. Another study in participants with T2DM reported that steviol glycoside reduced postprandial blood glucose without a significant change in insulin levels [148]. Furthermore, steviol glycoside was also shown to significantly reduce food intake [147]. Peirera et al. [149] did not demonstrate any effect of steviol glycoside on gastric emptying of a liquid test meal in 10 male healthy volunteers. Most of the studies in the literature have evaluated the effect of long-term steviol glycoside intake on BP and demonstrated that it may be effective in decreasing SBP and DBP compared with placebo in hypertensive [150,151], but not in normotensive or hypotensive, participants [152,153]. However, there have been no publications evaluating the acute effect of this sweetener on postprandial BP. 

### 5.6. Neotame and Advantame

Neotame is closely related to aspartame in terms of the chemical constitution. It is 7000–13,000 times sweeter than sucrose [154]. Advantame is considered the most potent non-caloric sweetener with sweetness approximately 20,000 times greater than sucrose [154]. Advantame is derived from isovanillin and aspartame [155]. To our knowledge there have been no studies evaluating the effects of these sweeteners on BP.

## 6. Conclusions 

PPH is associated with an increased incidence of falls, syncope, angina and transient ischaemic attacks, particularly in older people and patients with autonomic failure that is frequently secondary to diabetes mellitus and PD. For nutritive sweeteners, glucose induces the greatest fall in postprandial BP and should be limited in people with PPH. Most of the currently available literature indicates that non-nutritive sweeteners have little effect on BP. While pharmacological and dietary approaches are being explored, current management of PPH remains suboptimal. Our literature review supports the view that low nutritive (d-xylose, xylitol, erythritol, maltose, maltodextrin, and tagatose) and non-nutritive sweeteners could be used to replace high nutritive sweeteners (glucose, fructose, and sucrose) in the dietary management of PPH.

## Figures and Tables

**Table 1:** Studies relating to the effects of glucose on blood pressure.

Study	Year	Participant Characteristics	Study Design	Test Meal	Effects on Blood Pressure
Borg et al. [38]	2019	10 diet controlled T2DM patients, 5M:5F, aged 65.6 ± 3.1 years	Randomized crossover study	ID metformin (1 g) or saline (control) 60 min before ingesting a 50 g glucose drink labelled with 150 mg 13C-acetate.	SBP and DBP decreased following oral glucose on both days. The fall in SBP was less after metformin than control.
Brown et al. [39]	2008	15 healthy normal-weight participants, 9M:6F, aged 24 ± 1 years	Randomized crossover study	500 mL of either water, 60 g glucose, or 60 g fructose.	Oral fructose, but not glucose, significantly increased SBP and DBP. The maximum rise in SBP after fructose was 6.2 ± 0.8 mmHg.
Charriere et al. [89]	2017	9 young healthy men, aged 24 ± 1 years	Randomized crossover study	500 mL of water containing 60 g of either glucose, fructose or galactose.	The increase in SBP after fructose (7–8 mmHg) was greater than after glucose (4–5 mmHg) or galactose (2–3 mmHg). DBP increased to a greater extent with fructose (~5 mmHg), compared to non-significant increases of only 2–3 mmHg after glucose or galactose.
Edwards et al. [51]	1996	10 young (20–40 years), 9 middle-aged (41–50 years), and 10 old (61–83 years) participants	Non-randomized study	75 g glucose in 300 mL water	SBP decrease was significant in both the older groups. A fall in SBP > 15 mmHg observed in 5 individuals; 4 aged >60 years and 1 middle aged.
Fagius et al. [52]	1994	39 participants in 5 groups: glucose (*n* = 8, 4M:4F, mean age 25.8 years), fat (*n* = 8, 5M:3F, mean age 25.5 years), protein (*n* = 8, 5M:3F, mean age 25.6 years), mixed meal (*n* = 8, 6M:2F, mean age 26.2 years) or water (*n* = 7, 4M:3F, mean age 24.9 years).	Parallel study	100 g glucose in 300 mL of water (*n* = 8), 50 g fat in 250 mL of water (*n* = 8), 100 g lean meat (40 g protein) with 250 mL water (*n* = 8), 300 mL water alone (*n* = 7) or a mixed meal (*n* = 8).	Small and sometimes significant increases in BP occurred during the sessions.
Fagius et al. [53]	1996	3 groups—A: 9 young healthy, 5M:4F, aged 26.2 ± 2.8 years; B: 9 older healthy, all M, aged 73.0 ± 0.7 years; C: 9M with insulin resistance aged 72.8 ± 1.6 years	Non-randomized study	100 g D-glucose in 300 mL	Significant fall in BP observed in groups B and C but not in group A, who demonstrated an increase in SBP.
Gentilcore et al. [74]	2006	8 healthy older participants, 3M;5F, aged 65–78 years	Randomized crossover study	50 g glucose in either 300 mL (16.7%), 600 mL (8.3%), or 1200 mL (4.1%) or saline (0.9%) at a similar rate of 3 kcal/min	SBP and DBP decreased, and HR increased, on all days following the glucose infusions with no difference between them.
Gentilcore et al. [83]	2007	10 healthy older participants, 5M:5F, aged 65–76 years	Randomized crossover study	Granisetron (10 mcg/kg) or control (saline) at *t* = −25 min; ID glucose infusion (3 kcal/min) for 60 min, followed by ID saline for a further 60 min.	There were falls in SBP and DBP and a rise in HR during ID glucose; granisetron had no effect on these responses.
Gentilcore et al. [14]	2008	8 healthy older participants, 4M:4F, aged 68–79 years	Randomized crossover study	ID glucose (64 g), fat (10% oil emulsion), protein (72 g whey), or saline (0.9%) infusion at a rate of 2.7 mL/min for 90 min, followed by ID saline for 30 min	The maximum falls in SBP during the glucose (11.7 ± 2.8 mmHg), fat (11.7 ± 4.8 mmHg), and protein (11.0 ± 1.5 mmHg) infusion did not differ significantly. The fall occurred significantly earlier during the glucose (18 ± 3 min) than during the fat (46 ± 11 min) and protein (33 ± 7 min) infusion.
Gentilcore et al. [75]	2008	8 healthy older participants, 5M:3F, aged 65–76 years	Randomized crossover study	(1) ID glucose (50 g) or (2) ID glucose (50 g) with intragastric infusion of 500 mL water or (3) ID saline (0.9%) with intragastric infusion of 500 mL water.	The fall in SBP and DBP greater during (1) and (2) when compared with (3) and (1) compared with (2). Gastric distension attenuated the fall in BP.
Gentilcore et al. [54]	2009	8 healthy participants, 5M:3F, aged 66–75 years	Randomized crossover study	Day 1: ingestion of 75 g glucose in 300 mL. Gastric emptying rate (kcal/min) quantified by 3D ultrasound between *t* = 0–120 min. Day 2: ID glucose infused at the same rate as day 1.	SBP was greater less after oral, compared with ID glucose.
Grasser et al. [40]	2014	12 healthy young adults, 7M:5F, aged 22.0 ± 0.4 years	Randomized crossover study	500 mL drink of either 60 g sucrose, 60 g glucose, 60 g fructose or 30 g fructose.	Ingestion of fructose (60 or 30 g) elevated SBP, DBP and mean arterial pressure (MAP). Ingestion of glucose elevated SBP. Ingestion of sucrose showed no BP changes. The increases in DBP and MAP were significantly higher for fructose (60 or 30 g) than for either glucose or sucrose. The increase in SBP was significantly higher for fructose than for sucrose.
Heseltine et al. [55]	1991	20 older adults, 10M:10F, aged 84 ± 5 years	Randomized crossover study	400 kcal glucose drink with either caffeinated coffee or decaffeinated coffee	Maximal postprandial fall in sitting SBP was attenuated by caffeine. Four participants developed symptomatic PPH after placebo which was prevented by caffeine.
Hirayama et al. [56]	1993	10 patients with MSA, 9M:1F, aged 57 ± 7 years, 3 patients with peripheral autonomic neuropathy, 2M,1F, aged 35–57 years and 16 controls, 14M:2F, aged 38 ± 11 years.	Non-randomized study	75 g glucose in 225 mL water	In MSA, ingestion of glucose resulted in a rapid and significant fall of SBP and DBP. In peripheral autonomic neuropathy, BP decreased within 15 min of oral glucose ingestion, but soon recovered. BP in the controls remained unchanged.
Hirayama et al. [56]	1993	5 patients with MSA, 3M:2F, aged 50–71 years, 2 patients with pure autonomic failure, 2M: 54–78 years and 1 71-year-old F patient with autonomic failure and Parkinson’s disease. All with PPH and OH	Crossover study	Denopamine and midodrine administered 30 min before 75 g glucose drink on one day versus no drug a few days prior.	PPH was prevented by denopamine and midodrine.
Hoeldtke et al. [58]	1989	6 MSA patients, 4M:2F, aged 53–73 years, 5 progressive autonomic failure patients, 3M:2F, aged 41–84 years) and 14 controls, 9M:2F, aged 36–89 years.	Crossover study	SMS-201–995 (0.8 mcg/kg) or placebo injection sc before consuming a 50 g glucose drink.	In patients with progressive autonomic failure and MSA, glucose ingestion caused a decrease in BP which was attenuated by SMS-201–995.
Jansen et al. [41]	1987	10 young normotensive people, aged 28 ± 1 years (YN), 10 young hypertensive patients, aged 44 ± 2 years (YH), 10 elderly normotensive people aged 75 ± 2 years (EN), 10 elderly hypertensive patients aged 75 ± 1 years (EH).	Randomized crossover study	300 mL drink of either 75 g glucose or 75 g fructose.	Glucose decreased MAP significantly in the EH, EN and YH group. After fructose, BP remained unchanged in 4 groups.
Jansen et al. [59]	1988	Hypertensive patients: randomised to nitrendipine: 4M:5F, aged 70–78 years—or hydrochlorothiazide: 3M:10F, aged 70–84 years	Randomized parallel study	75 g glucose drink before and after treatment with 20 mg nitrendipine once daily or 50 mg hydrochlorothiazide once daily for 12 weeks.	After 12 weeks of treatment, nitrendipine reduced the fall in MAP after oral glucose (6%, *p* < 0.01) but this was not significant for hydrochlorothiazide (4%, NS).
Jansen et al. [60]	1989	10 hypertensive participants, 3M:7F, aged 74 ± 4 years; and 10 normotensive participants, 4M:6F, aged 74 ± 4 years	Randomized crossover study	Octreotide (50 mcg sc) or placebo (154 mmol/L NaCl) before oral 75 g glucose in 300 mL water	Octreotide attenuated the fall in MAP (15 ± 1 mmHg in the 10 hypertensive participants and 7 ± 2 mmHg in the 10 normotensive participants) induced by oral glucose.
Jansen et al. [61]	1989	10 hypertensive participants, 7M:3F, aged 73 ± 3 years	Randomized crossover study	Octreotide (50 mcg sc) at *t* = −30 min followed by placebo or insulin (0.3 U/kg) sc at *t* = −10 min and oral glucose (75 g in 300 mL water) at *t* = 0 min	The fall in MAP after oral glucose was attenuated by octreotide with no difference between the insulin and placebo study days.
Jansen et al. [62]	1989	15 older hypertensives (EH), 7M:8F, age 73 ± 3 years, 15 older normotensives (EN), 6M:6F, age 76 ± 4 years and 10 young normotensives (YN), 5M:5F, age 26 ± 4 years.	Non-randomized study	75 g glucose in 300 mL water	In both elderly groups MAP decreased significantly after the glucose load, whereas no change was observed in the YN. Glucose load did not influence baroreflex sensitivity.
Jones et al. [11]	1998	16 T2DM patients, 11M:5F, aged 39–79 years; 10 young healthy participants, 9M:9F, aged 19–26 years; 9 older healthy participants, 6M:3F, aged 40–68 years old.	Non-randomized study	75 g glucose in 350 mL water	The fall in MAP was significantly greater in the T2DM than in older healthy participants with no change in young healthy participants. The magnitude of the fall in BP was related to the rate of gastric emptying.
Jones et al. [11]	2005	10 healthy participants, 6M:6F, aged 73.9 ± 1.2 years	Randomized crossover study	25 g glucose in 200 mL (12.5%), 75 g glucose in 200 mL (37.5%), 25 g glucose in 600 mL (4%), and 75 g glucose in 600 mL (12.5%)	Increased drink volume attenuates the fall in BP with no effect of glucose concentration.
Jones et al. [63]	2001	10 healthy participants, 5M:5F, aged 67–78 years	Randomized crossover study	300 mL water containing 50 g glucose with 30 mL lemon juice made up to 300 mL with or without 9 g guar gum	SBP, DBP and MAP fell on both days. The magnitude of the falls in SBP, DBP, and MAP were less, after guar.
Jones et al. [64]	2019	15 healthy participants, 9M:6F, aged 67.2 ± 2.3 years and 15 T2DM patients, 9M:6F, aged 61.9 ± 2.3 years)	Randomized crossover study	Lixisenatide (10 mcg) or placebo sc 30 min before 75 g glucose drink on two separate days.	Lixisenatide attenuated the decrease in SBP and DBP compared to placebo in healthy participants and those with T2DM
Marathe et al. [76]	2016	9 patients with T2DM, all M, aged 62 ± 2.4 years	Randomized crossover study	ID glucose (25 g/100 mL) infused at 2 kcal/min or 4 kcal/min	SBP and DBP fell at 30 min with 4 kcal/min, but not 2 kcal/min infusions. The fall in SBP was greater after the 4 kcal/min infusion.
Maruta et al. [76]	2006	28 neurologic patients (11 with PD, 4M:7F, aged 61–86 years; 6 with MSA, 4M:2F, aged 53–76 years; 11 with T2DM, 8M:3F, aged 62–85 years) and 20 healthy controls (13 older participants, 5M:8F, aged 62–80 years; 7 young participants, 4M:3F, aged 34–59 years).	Crossover study	75 g glucose with or without 200 mcg voglibose. All participants were studied on the day without voglibose. 11 of them (4 with PD, 5 with MSA, 1 with T2DM, 1 older control), who had PPH, were studied on the day with voglibose.	The fall in BP was less (without voglibose: 41.5 ± 13.2 mmHg, with voglibose: 21.0 ± 13.0 mmHg) and the duration of PPH was shorter (without voglibose: 52.3 ± 28.0 min, with voglibose: 17.3 ± 22.5 min) after voglibose.
Masuo et al. [66]	1996	12 young normotensive (NT) participants, aged 47.8 ± 2.6 years; 21 elderly NT, aged 77.9 ± 1.5 years; 17 young hypertensive (EH) patients, aged 49.0 ± 1.9 years and 32 elderly EH. 1M:1F in each group.	Non-randomized study	75 g glucose in 225 mL water	Postprandial BP reduction, defined as 10% or more decline in MAP was recognized in 3/12 (25%) young NT, 9/21 (43%) elderly NT, 5/17 (29%) young EH, and 20/32 (63%) elderly EH. The frequency of postprandial BP reduction was significantly greater in elderly hypertensives compared to elderly normotensives and was greater in young hypertensives compared to young normotensives.
Mathias et al. [43]	1989	6 patients with chronic autonomic failure (CAF), 4M:2F, aged 42–68 years, 6 age-matched participants without CAF, aged 45–70 years; and 8 normal participants, all M, aged 28–35 years.	Randomized parallel study	An iso-osmotic solution of glucose (1 g/kg body weight) or xylose (0.83 g/kg body weight) in 250 mL water. 6 patients with CAF attended 2 on both glucose and xylose days. 6 age-matched participants and 8 male normal participants attended only on the glucose day.	Xylose caused a lower and more transient fall in BP than glucose in patients with CAF (15 ± 6% vs. 34 ± 7%). After glucose, there was a substantial fall in 6 age-matched participants but a minimal change in 8 male normal participants.
Nair et al. [67]	2015	13 participants with PPH, 4M:9F, aged 76.5 ± 4 years.	Randomized crossover study	Ingestion of 50 g glucose in 200 mL on both days. On one day, participants walked at their usual pace for 30 m every 30 min for 120 min.	On the control day, there were significant falls in SBP and DBP. On the intervention day, there was no significant fall in SBP, however, DBP still fell significantly.
Nair et al. [68]	2016	29 older participants, 18F aged 77.1 ± 5.4 years and 11M aged 74.7 ± 3.9 years	Randomized crossover study	3 treatments: glucose (50 g in 200 mL) (G) or water (200 mL) and intermittent walking (WW) or glucose and walking (GW)	16 participants had PPH. In PPH, there was a significant fall in SBP (26.69 ± 8.43 mmHg) on the “G” day and no change on “GW” or “WW” days. In those without PPH, there were no changes in SBP on the “G” or “GW” days, with an increase in SBP on the “WW” day.
Nguyen et al. [44]	2018	35 older participants, 28M:7F, aged 73 ± 5 years, discharged at least 3 months from ICU	Non-randomized study	300 mL drink containing 75 g glucose	There were significant reductions in both SBP and DBP. Ten participants (29%) had postprandial hypotension. The maximal fall in SBP and DBP were −29 ± 14 mmHg and −18 ± 7 mmHg. The maximal fall in SBP was greater in patients with PPH than in those without (−46.2 ± 10.8 mmHg vs. −22.7 ± 9.2 mmHg).
O’Donovan et al. [77]	2002	8 healthy elderly participants, 4M:4F, aged 70.3 ± 3.4 years	Randomized crossover study	25% glucose solution was infused intraduodenally at a rate of either 1 or 3 kcal/min for 60 min followed by 0.9% saline for a further 60 min	Between t = 0–60 min, there was a fall in SBP, DBP and MAP during the 3 kcal/min glucose infusion, but not during the 1 kcal/min infusion.
O’Donovan et al. [84]	2005	8 healthy older participants, 4M:4F, aged 70.3 ± 3.4 years	Randomized crossover study	ID glucose infusion (3 kcal/min) with or without guar gum (4 g) for 60 min, followed by 0.9% saline intraduodenally for a further 60 min.	Between t = 0–60 min, SBP was lower during the glucose-only infusion than during the glucose and guar infusion. The maximum fall in SBP on the glucose-only study was 10 ± 4 mmHg. Between *t* = 0–30 min, DBP fell during the glucose-only infusion, but did not change with the glucose and guar infusion.
Pham et al. [78]	2018	12 healthy participants, 6M:6F, aged 73.2 ± 1.1 years	Randomized crossover study	ID infusion of either glucose (25%, ~1400 mOsmol/L), sucralose (4 mmol/L, ~300 mOsmol/L) or saline (0.9%, ~300 mOsmol/L) at a rate of 3 mL/min for 60 min followed by ID saline for a further 60 min.	MAP decreased during glucose but not during sucralose or saline. By *t* = 60 min, MAP was lower after glucose (85.9 ± 2.8 mmHg) than after sucralose (93.1 ± 2.2 mmHg) infusions without significant difference between sucralose and saline infusions.
Pham et al. [12]	2019	33 healthy older participants, 16M:17F, aged 77.0 ± 0.7 years	Non-randomized study longitudinal study	75 g glucose in 300 mL water	The prevalence of PPH doubled from 9.1% to 18.2%. There was a fall in SBP and DBP on both study days. The AUC of SBP was greater at follow-up. The maximum fall in postprandial SBP between *t* = 60–120 min was significantly greater at follow-up (−11.7 ± 1.4 vs. −15.2 ± 1.6 mmHg).
Robinson et al. [45]	1992	5 participants with age-related OH, 2M:3F, aged 73–88 years), 3 participants with autonomic failure, 1M:2F, aged 72–79 years and 5 controls, 2M:3F, aged 72–86 years	Randomized crossover study	50 g glucose or 42 g xylose in 100 mL water	In OH and autonomic failure groups, the SBP decreased comparably following glucose and xylose, DBP was lowered 60–90 min after glucose. No significant BP changes in the control group.
Russo et al. [13]	2003	11 patients with T2DM managed by diet alone, 8M:3F, aged 61.9 ± 1.3 years	Randomized crossover study	50 g glucose and 30 mL lemon juice, with or without 9 g guar gum in 300 mL.	There was significant fall in SBP between baseline and 30 min on the control day (143.9 ± 4.7 mmHg vs. 139.0 ± 4.2 mmHg; *p* < 0.01), but not after guar (145.1 ± 4.8 mmHg vs. 142.6 ± 4.5 mmHg, *p* = 0.6). There were significant falls in DBP and MAP between baseline and 30 min on both study days.
Sasaki et al. [69]	1992	15 normal participants, aged 25–63 years and 35 outpatients with T2DM, aged 28–60 years	Non-randomized study	Daily meals and 75 g glucose in 300 mL water	No significant change in BP in the normal participants. The incidence of PPH in diabetics was 37% after daily meal and 20% after 75 g glucose.
Takamori et al. [70]	2007	17 MSA patients, 9M:8F, aged 59.8 ± 7.9 years and 8 healthy controls, 7M:1F, aged 60.5 ± 8.3 years	Non-randomized study	75 g of glucose in 225 mL of water	Of 17 MSA patients, 9 had PPH. 8 controls were PPH negative. The falls in SBP and DBP in MSA with PPH were significantly greater than in MSA without PPH or in controls.
Thazhath et al. [85]	2017	9 patients with T2DM, managed by diet alone, 6M:3F, aged 60.7 ± 2.4 years	Randomized crossover study	Intravenous exenatide (7.5 mcg) or volume-matched saline control from −30 to 120 min + ID glucose (3 kcal /min) from 0–60 min.	During the ID glucose infusion, SBP, DBP and MAP increased with exenatide, but fell with saline control. The AUC for DBP and MAP, but not SBP, was higher with exenatide than control.
Trahair et al. [16]	2015	14 older healthy participants, 6M:8F, aged 72.1 ± 1.1 years and 10 patients with T2DM, 6M: 4F, aged 68.7 ± 3.4 years	Randomized crossover study	Between *t* = −30–120 min: intravenous infusion of GLP-1 (0.9 pmol/kg/min) or saline (154 mmol/l NaCl). At *t* = 0 min: 75 g glucose drink in 300 mL water	After the glucose drink there were falls in SBP and DBP in both groups. The fall in DBP in older individuals; and the fall in SBP and DBP in patients with T2DM were less after GLP-1 infusion compared to control.
Trahair et al. [79]	2012	12 healthy young participants, 6M;6F, aged 22.2 ± 2.3 years and 12 healthy older participants, 6M; 6F, aged 68.7 ± 1.0 years	Randomized crossover study	ID infusion of glucose at either 1, 2 or 3 kcal/min or 0.9% normal saline for 60 min followed by ID saline for a further 60 min.	In young participants, there were no changes in SBP and DBP during the four infusions. In older participants, there were falls in SBP and DBP during 2 kcal/min and 3 kcal/min infusions, but not during 1 kcal/min infusion.
Trahair et al. [18]	2014	10 healthy older participants, 9M: 1F, aged 73.2 ± 1.5 years	Randomized crossover study	Between *t* = −30–60 min, intravenous infusion of GLP-1 (0.9 pmol/kg/min), or saline for 90. Between *t* = 0–60 min, ID glucose was infused at 3 kcal/min.	During ID glucose infusion, there were falls in SBP and DBP with both GLP-1 and control. The maximum fall in SBP was greater with control than GLP-1 (−13.6 ± 3.1 mmHg vs. −8.7 ± 2.3 mmHg).
Trahair et al. [46]	2015	88 healthy older participants, 41M:47F, aged 71.0 ± 0.5 years	Non-randomized study	75 g glucose in 300 mL water	SBP and DBP decreased significantly after the glucose drink. Eleven participants (12.8%) had PPH.
Trahair et al. [47]	2016	21 participants with mild to moderate PD, 13M:8F, aged 64.2 ± 1.6 years	Crossover study	75 g glucose in 300 mL water	SBP and DBP fell following the glucose drink. 8 participants (38%) had postprandial hypotension.
Trahair et al. [48]	2017	8 healthy older participants, 4M:4F, aged 71.0 ± 1.7 years and 8 participants with PPH 1M:7F, aged 75.5 ± 1.0 years	Randomized crossover study	75 g glucose in 300 mL water or water alone	Following the glucose, there were decreases in SBP and DBP in both groups, the maximum fall in SBP was greater in participants with PPH. Following the water, there were no changes in SBP and DBP in healthy participants, but there was a rise in SBP in participants with PPH.
Trahair et al. [80]	2018	12 obese participants, 10M:2F, aged 36.6 ± 3.9 years, BMI: 36.1 ± 1.3 kg/m^2^) and 23 controls, 16M:7F, aged 27.8 ± 2.4 years, BMI: 22.4 ± 0.5 kg/m^2^	Randomized crossover study	ID infusions of glucose at 1 or 3 kcal/min, or 0.9% saline, for 60 min, followed by saline for a further 60 min.	No changes in SBP in both groups during any of the conditions. There was a fall in DBP in controls during 1 kcal/min and 3 kcal/min infusions; and in obese participants during 3 kcal/min infusion. There was no difference in BP responses between the groups.
Umehara et al. [71]	2014	37 patients with de novo PD (17 with PPH, 4M:13F, aged 76.8 ± 6.1 years; 20 without PPH, 8M:12F, aged 74.4 ± 7.5 years) and 10 healthy controls, aged 74.3 ± 4.8 years)	Non-randomized study	75 g glucose in 300 mL water	Of the 37 patients, 17 (45.9%) had PPH, 15 (40.5%) had OH and 8 (21.6%) had both PPH and OH. 2 controls had PPH. The maximum fall in SBP after the glucose drink significantly correlated with that on head-up tilt-table testing in PD patients.
Umehara et al. [72]	2016	64 de novo patients with PD, 22M:42F, aged 76 ± 4 years	Non-randomized study	75 g glucose in 300 mL water	29 patients had PPH. Patients with PPH experienced greater reductions in SBP (30 ± 11 vs. 11 ± 15 mmHg) and DBP (14 ± 9 vs. 7 ± 5 mmHg) after glucose drink compared to patients without PPH.
van Orshoven et al. [81]	2008	8 healthy young participants (4M:4F, aged 28.8 ± 3.4 years), 8 healthy elderly (4M:4F, aged 75.3 ± 1.6 years). 2 female patients with symptomatic PPH aged 21 and 90 years	Non-randomized study	ID infusion of 25% glucose at 3 mL/min for 60 min. Saline was infused for 30 min before and after ID glucose.	ID glucose decreased SBP, in both the young and older people, but the fall in SBP was greater in the older group (−6.5 ± 1.6 vs. −17.0 ± 4.1 mmHg). 2 PPH patients had a greater fall in SBP than the two healthy groups (−21 and −98 mmHg).
Vanis et al. [82]	2011	12 healthy older participants, 6M:6F, aged 68.7 ± 1.0 years	Randomized crossover study	ID infusion of glucose at either 1, 2 or 3 kcal/min or 0.9% normal saline for 60 min, followed by saline for a further 60 min.	There was a fall in SBP and DBP during 2 and 3 kcal/min glucose infusions, but not during saline or 1 kcal/min glucose infusion. There was no difference in the maximum falls in SBP during 2 kcal/min (15 ± 2 mmHg) and 3 kcal/min (12 ± 2 mmHg) loads.
Vanis et al. [49]	2011	8 healthy older participants, 6M:2F, aged 65–75 years	Randomized crossover study	300 mL drink of water, 50 g glucose or 50 g d-xylose.	There was a fall in SBP after glucose drink and no change after xylose or water drink.
Vanis et al. [86]	2010	8 participants, 6M:2F, aged 65–75 yeas	Randomized crossover study	The four treatments were as follows: ID glucose (3 kcal/min) + barostat (distension) (GD), ID saline + barostat (SD), ID glucose (G), and ID saline (S).	SBP and DBP fell during G, but not during S or GD; and increased during SD. The maximum changes in SBP during G, GD, S and SD were −14 ± 5, −3 ± 4, +11 ± 2, and +15 ± 3 mmHg respectively.
Vanis et al. [87]	2012	9 participants, all M, aged 65–75 years	Randomized crossover study	ID glucose infusion (3 kcal/min) and gastric distension at a volume of (1) 0 ml (V0), (2) 100 mL (V100), (3) 300 mL (V300), or (4) 500 mL (V500).	SBP and DBP fell during V0, but did not change significantly during V100, V300, V500.
Visvanathan et al. [73]	2006	12 elderly participants, 6M:6F, aged 72.2 ± 5.7 years	Randomized crossover study	300 mL drink of either (1) CHO (75 g glucose and 93 g Polyjoule (CHO polymer)-653 kcal); (2) 88% fat (cream blended with milk-653 kcal) or (3) water.	SBP decreased following the CHO drink and the high-fat drink but not water; there was no difference in the magnitude of the decrease between the CHO and fat drinks. The onset of the SBP fall was slower after the fat drink (26.5 ± 17.1 min vs. 13.0 ± 11.7 min).
Visvanathan et al. [50]	2005	10 healthy older participants, 4M; 6F, aged 72.2 ± 1.50 years	Randomized crossover study	300 mL of either 50 g glucose, 50 g sucrose, 50 g fructose or water + 30 mL lemon juice	SBP decreased significantly following glucose (−3.96 ± 1.38 mmHg) and sucrose (−3.03 ± 1.37 mmHg) ingestion, increased non-significantly following fructose ingestion (2.59 ± 1.62 mmHg). The decrease in SBP occurred earlier after glucose than sucrose ingestion (7.33 ± 2.19 vs. 21.0 ± 4.30 min).
Wu et al. [88]	2017	Study A: 16 participants with T2DM, 11M: 5F, 65.5 ± 2.4 years. Study B: 9 participants with T2DM, all M, aged 63.8 ± 2.6 years	Randomized crossover study	Study A: vildagliptin (50 mg) or placebo was given 60 min before ID glucose infusion at 2 or 4 kcal/min (ID2 or ID4). Study B: Participants received metformin (850 mg) or placebo for 7 days. On the study day, metformin (850 mg) or placebo was given 30 min before ID2.	Study A: SBP and DBP decreased after vildagliptin, but not after placebo, without any difference between ID2 and ID4. Study B: SBP and DBP decreased on both days without any difference between metformin and placebo.

T2DM = type 2 diabetes; BP = blood pressure; SBP = systolic blood pressure; DBP = diastolic blood pressure; HR = heart rate; ID = intraduodenal; MAP = mean arterial pressure; PD = Parkinson’s disease; MSA = multi-system atrophy; PPH = postprandial hypotension; OH = orthostatic hypotension; CHO = carbohydrate; GLP-1 = glucagon-like peptide-1; AUC = area under the curve; NS = not significant; M = male; F = female.

**Table 2:** Studies relating to the effects of other nutritive sweeteners on blood pressure.

Study	Year	Participant Characteristics	Study Design	Sweeteners Assessed rather than Glucose	Test Meal	Effects on Blood Pressure
Brown et al. [39]	2008	15 healthy normal-weight volunteers, 9M:6F, aged 24 ± 1 years	Randomized crossover study	Fructose	500 mL of either water, 60 g glucose, or 60 g fructose.	Oral fructose, but not glucose, significantly increased SBP and DBP. The maximum rise in SBP after fructose was 6.2 ± 0.8 mmHg.
Charriere et al. [89]	2017	9 young healthy men, aged 24 ± 1 years	Randomized crossover study	Galactose, fructose	500 mL of water containing 60 g of either glucose, fructose or galactose.	The increase in SBP after fructose (7–8 mmHg) was greater than after glucose (4–5 mmHg) or galactose (2–3 mmHg). DBP increased to a greater extent with fructose (~5 mmHg), compared to non-significant increases of only 2–3 mmHg after glucose or galactose.
Gentilcore et al. [105]	2005	8 healthy older participants, 5M:3F, aged 65–79 years	Randomized crossover study	Sucrose	300 mL drink of 100 g sucrose and 30 mL lemon juice with or without 100 mg acarbose	There was a fall in SBP and DBP on the control day while there was an overall increase in SBP on the acarbose day.
Gentilcore et al. [106]	2011	8 healthy older participants, 4M:4F, aged 66–77 years	Randomized crossover study	Sucrose	ID infusion of sucrose (100 g/300 mL) at ~6 kcal/min with or without acarbose (100 mg), over 60 min.	There were significant falls in SBP (maximum fall: 11.2 ± 2.0 mmHg) during control, but not after acarbose. The fall in DBP was greater after control (10.9 ± 0.9 mmHg) than after acarbose (8.1 ± 1.5 mmHg).
Grasser et al. [40]	2014	12 healthy young adults, 7M:5F, aged 22.0 ± 0.4 years	Randomized crossover study	Fructose, sucrose	500 mL drink of either 60 g sucrose, 60 g glucose, 60 g fructose or 30 g fructose.	Ingestion of fructose (60 or 30 g) elevated SBP, DBP and MAP. Ingestion of glucose elevated SBP. Ingestion of sucrose showed no BP changes. The increases in DBP and MAP were significantly higher for fructose (60 or 30 g) than for either glucose or sucrose. The increase in SBP was significantly higher for fructose than for sucrose.
Jansen et al. [41]	1987	10 young normotensive people, aged 28 ± 1 years (YN), 10 young hypertensive patients, aged 44 ± 2 years (YH), 10 elderly normotensive people aged 75 ± 2 years (EN), 10 elderly hypertensive patients aged 75 ± 1 years (EH)	Randomized crossover study	Fructose	300 mL drink of either 75 g glucose or 75 g fructose	Glucose decreased MAP significantly in the EH, EN and YH group. After fructose, BP remained unchanged in 4 groups.
Mathias et al. [43]	1989	6 patients with chronic autonomic failure (CAF), 4M:2F, aged 42–68 years; 6 age-matched participants without CAF, aged 45–70 years; and 8 normal participants, all M, aged 28–35 years	Randomized parallel study	Xylose	An iso-osmotic solution of glucose (1 g/kg body weight) or xylose (0.83 g/kg body weight) in 250 mL water.	Xylose caused a lower and more transient fall in BP than glucose in patients with CAF (15 ± 6% vs. 34 ± 7%). After glucose, there was a substantial fall in 6 age-matched participants but a minimal change in 8 male normal participants.
Robinson et al. [45]	1992	5 people with age-related OH, 2M:3F, aged 73–88 years, 3 people with autonomic failure, 1M:2F, aged 72–79 years and 5 controls, 2M:3F, aged 72–86 years	Randomized crossover study	Xylose	50 g glucose or 42 g xylose in 100 mL water	There were no significant BP changes in the control group. In OH and autonomic failure groups, the SBP decreased comparably following glucose and xylose, DBP was lowered 60–90 min after glucose.
Teunissen-Beekman et al. [107]	2014	48 participants, 31M; 17F, aged 58 ± 1 (SEM) years	Randomized crossover study	Maltodextrin, sucrose	Test drink of 70 g either protein (pea protein isolate, milk protein isolate, egg white protein isolate or mixed protein), sucrose or maltodextrin.	DBP and MAP were significantly decreased after maltodextrin, but not after protein mix or sucrose. SBP was not significantly changed after any of the meals.
Vanis et al. [49]	2011	8 healthy older participants, 6M:2F, aged 65–75 years	Randomized crossover study	Xylose	300 mL drink of water, 50 g glucose or 50 g d-xylose.	There was a fall in SBP after glucose drink and no change after xylose or water drink.
Visvanathan et al. [50]	2005	10 healthy older participants, 4M:6F, aged 72.2 ± 1.50 years	Randomized crossover study	Fructose, sucrose	300 mL of either 50 g glucose, 50 g sucrose, 50 g fructose or water + 30 mL lemon juice	SBP decreased significantly following glucose (−3.96 ± 1.38 mmHg) and sucrose (−3.03 ± 1.37 mmHg), but not fructose, ingestion (2.59 ± 1.62 mmHg). The decrease in SBP occurred earlier after glucose than sucrose ingestion (7.33 ± 2.19 vs. 21.0 ± 4.30 min).

T2DM = type 2 diabetes; BP = blood pressure; SBP = systolic blood pressure; DBP = diastolic blood pressure; ID = intraduodenal; MAP = mean arterial pressure; MSA = multi-system atrophy; PPH = postprandial hypotension; OH = orthostatic hypotension.

**Table 3:** Studies relating to the effects of non-nutritive sweeteners on blood pressure.

Study	Year	Participant Characteristics	Study Design	Non-Nutritive Sweeteners Assessed	Test Meal	Effects on Blood Pressure
Kazmi et al. [140]	2017	200 students divided equally into 4 groups: A (aged 18.82 ± 0.80 years), B: (aged 18.60 ± 0.57), C (aged 18.64 ± 0.59) and D (18.64 ± 0.59)	Parallel study	Aspartame, Acesulfame-K, sucralose	Group A (control): 10 g of cellulose. Group B: 0.36 gm (5 mg/kg) sucralose. Group C: 10.8 g (150 mg/kg) aspartame. Group D: 3.24 g (45 mg/kg) Acesulfame-K.	There was no difference in BP between group A and B. SBP was lower at 60, 90 and 120 min for group C; and at 60 min for group D compared to control.
Pham et al. [78]	2018	12 healthy participants, 6M: 6F, aged 73.2 ± 1.1 (SEM) years	Randomized crossover study	Sucralose	ID infusion of either glucose (25%, ~1400 mOsmol/L), sucralose (4 mmol/L, ~300 mOsmol/L) or saline (0.9%, ~300 mOsmol/L) at a rate of 3 mL/min for 60 min followed by ID saline for a further 60 min.	MAP decreased during glucose but not during sucralose or saline. By *t* = 60 min, MAP was lower after glucose (85.9 ± 2.8 mmHg) than after sucralose (93.1 ± 2.2 mmHg) infusions without significant difference between sucralose and saline infusions.

BP = blood pressure; MAP = mean arterial pressure; ID = intraduodenal.
